# Use of Buffers in Specific Contexts: Highly Trained Female Athletes, Extreme Environments and Combined Buffering Agents—A Narrative Review

**DOI:** 10.1007/s40279-023-01872-7

**Published:** 2023-10-25

**Authors:** Amelia J. Carr, Alannah K. A. McKay, Louise M. Burke, Ella S. Smith, Charles S. Urwin, Lilia Convit, William T. Jardine, Monica K. Kelly, Bryan Saunders

**Affiliations:** 1 Centre for Sport Research, Deakin University, 221 Burwood Highway, Burwood, VIC 3125 Australia; 2 Mary MacKillop Institute for Health Research, Australian Catholic University, Melbourne, VIC Australia; 3 Applied Physiology and Nutrition Research Group, Rheumatology Division, Faculdade de Medicina FMUSP, School of Physical Education and Sport, Universidade de São Paulo, University of São Paulo, São Paulo, Brazil; 4 Institute of Orthopaedics and Traumatology, Faculty of Medicine FMUSP, University of São Paulo, São Paulo, Brazil

## Abstract

This narrative review evaluated the evidence for buffering agents (sodium bicarbonate, sodium citrate and beta-alanine), with specific consideration of three discrete scenarios: female athletes, extreme environments and combined buffering agents. Studies were screened according to exclusion and inclusion criteria and were analysed on three levels: (1) moderating variables (supplement dose and timing, and exercise test duration and intensity), (2) design factors (e.g., use of crossover or matched group study design, familiarisation trials) and (3) athlete-specific factors (recruitment of highly trained participants, buffering capacity and reported performance improvements). Only 19% of the included studies for the three buffering agents reported a performance benefit, and only 10% recruited highly trained athletes. This low transferability of research findings to athletes’ real-world practices may be due to factors including the small number of sodium citrate studies in females (*n* = 2), no studies controlling for the menstrual cycle (MC) or menstrual status using methods described in recently established frameworks, and the limited number of beta-alanine studies using performance tests replicating real-world performance efforts (*n* = 3). We recommend further research into buffering agents in highly trained female athletes that control or account for the MC, studies that replicate the demands of athletes’ heat and altitude camps, and investigations of highly trained athletes’ use of combined buffering agents. In a practical context, we recommend developing evidence-based buffering protocols for individual athletes which feature co-supplementation with other evidence-based products, reduce the likelihood of side-effects, and optimise key moderating factors: supplement dose and timing, and exercise duration and intensity.

## Key Points

**Table:** 

The current literature indicates a benefit from use of buffering agents in real-world performance efforts in only 21% of studies in highly trained females, 22% of studies in extreme environments and 9% of studies with combined buffering agents.
The low percentages of studies with performance benefit reported may have been associated with some of the limitations in the literature, which could be addressed by research focusing on (i) studies in highly trained females, which include quantification of menstrual cycle (MC) factors, (ii) buffering studies conducted at altitude with performance tests simulating real-world competitive events and (iii) the use of various combinations of buffering agents by highly trained and elite research participants.
To develop buffering protocols for individual athletes, we recommend a staged and systematic approach that identifies opportunities for co-supplementation with other evidence-based products, quantifies buffering capacity and side-effects where feasible, and optimises key moderating factors: supplement dose and timing, and exercise duration and intensity.

## Introduction

**Fig. 1:**
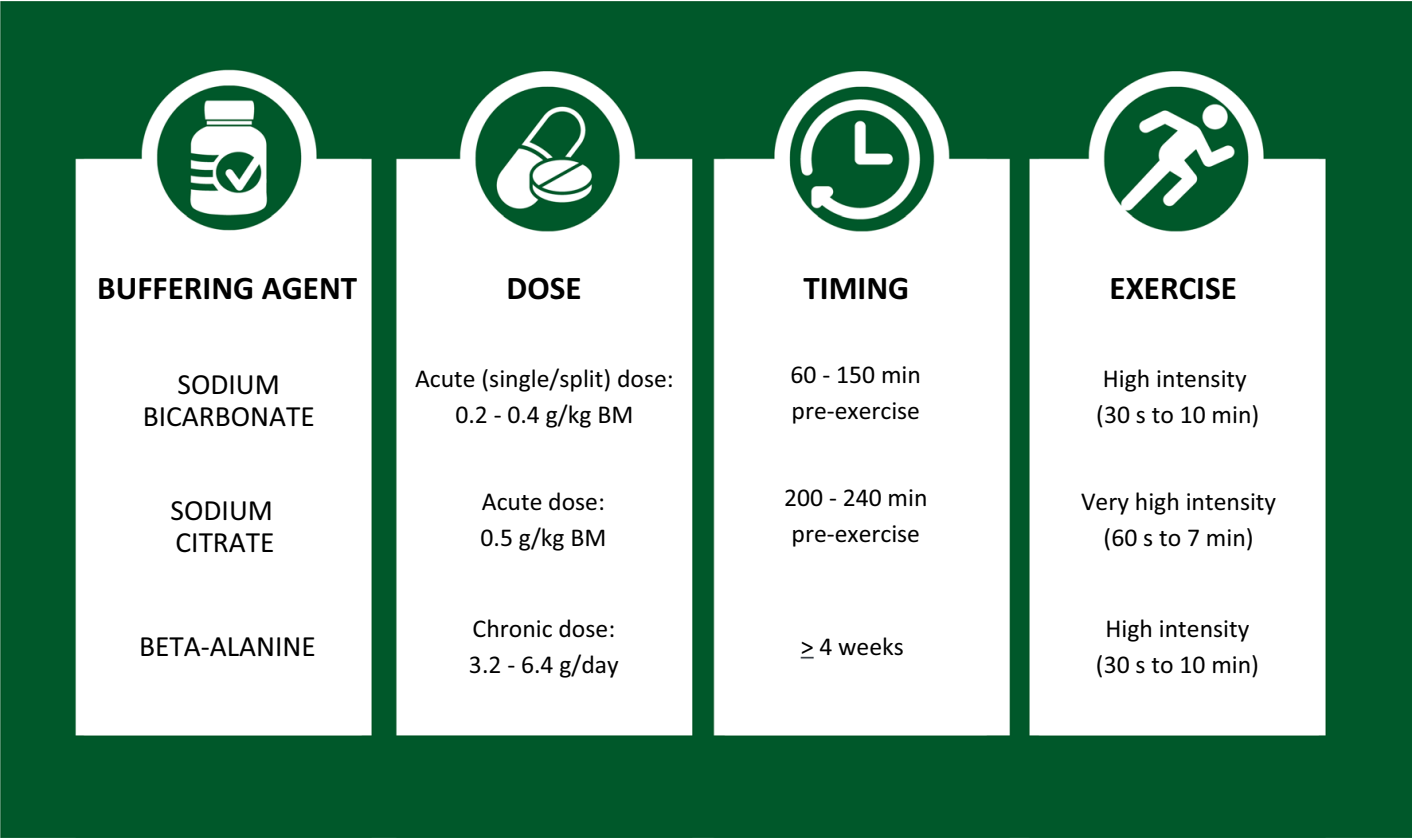
Current recommendations for buffering agents [1, 7]. *BM* body mass

The 2018 International Olympic Committee consensus statement on nutritional supplements concluded that five supplements have sufficient strength of scientific evidence to be considered effective in certain sport-specific scenarios [1]. Two of these supplements are buffering agents, namely beta-alanine and sodium bicarbonate. There are substantial meta-analytical data to support the performance-enhancing effects of both beta-alanine [2, 3] and sodium bicarbonate [4–6]. There is also growing evidence that sodium citrate can also be effective in improving exercise performance [5, 7]. Each of these supplements works by enhancing either intracellular or extracellular buffering capacity (increased blood bicarbonate concentration [HCO_3_^−^] and pH), with sodium bicarbonate and sodium citrate ingestion, or increasing muscle carnosine concentration with beta-alanine ingestion [3, 8]. Our knowledge of these supplements is continuously advancing, with novel scientific research filling important gaps that reshapes the recommendations provided to athletes. Current evidence-based recommendations focus on the dose and timing of supplement ingestion, and the duration and intensity of exercise that is most likely to benefit from these supplements; Fig. 1 [1, 7].

Within the body of literature investigating the effects of buffering agents, which began as early as the 1930s [9, 10], little consideration has been dedicated to their use in specific contexts that are relevant to the real-world practices of athletes. This includes the use of buffering agents in specific populations (e.g. highly trained female athletes) [11], extreme environmental conditions (e.g. training and/or competing in hot weather conditions (> 25 °C) or at altitude > 1400 m) [12–15], and specific supplementation practices (e.g. supplementation with combined buffering agents) [3, 16]. Consideration of the efficacy of buffering agents in these contexts is therefore warranted and can potentially be achieved by specific analysis of their study features and reported outcomes. Within the literature, it has recently been demonstrated that the effectiveness of extracellular and intracellular buffering agents can be influenced by several factors (as identified in previous meta-analyses and reviews of the literature), including the supplement dose and timing, and the duration and intensity of exercise being performed [3, 5, 7, 17–19]. Additionally, while moderating factors are typically based on the outcomes of meta-analytic and systematic reviews that include participants of varied training status and/or athletic experience, recent work has elucidated important study design factors that can increase external validity and positively influence the translation of sport science and sport nutrition studies to the real-world practices of athletes (e.g. use of randomised, crossover designs, incorporating exercise tests that are representative of real-world competitive events, conducting familiarisation trials and standardising pre-test meals) [20, 21]. Further, an increased specificity of analysis can be achieved through the consideration of factors that relate more directly to athletes’ use of buffering agents. These factors include the recruitment of highly trained athletes as research participants [i.e. according to the recently established participant classification framework] [22], and an increase in buffering capacity aligned with that reported to be consistent with performance benefits (i.e. ≥ 4 mmol/L blood [HCO_3_^−^] for extracellular buffering agents) [5]. For intracellular buffering agents, a similar threshold has not been established for the relationship between the increase in muscle carnosine concentration and performance enhancement. Nevertheless, a 40% increase in carnosine content is suggested as a minimum threshold for the increase associated with ingesting beta-alanine supplements at the lowest dose within the range of recommended protocols (i.e. 4 weeks at 3.2 g/day) [23], which is typically when performance benefits are shown. Further, a documented performance benefit, particularly in exercise tests that reflect real-world events, increases the relevance of research findings to athletes’ competitive performance [5, 21, 22, 24].

This narrative review will evaluate the current evidence supporting the use of buffering agents by athletes, with particular focus on their use in specific contexts (use by highly trained females, extreme environmental conditions and combined buffering agents). The narrative review will summarise (1) moderating factors, (2) translation factors and (3) athlete-specific factors in relation to the use of sodium bicarbonate, sodium citrate and beta-alanine. Finally, a detailed commentary will be provided on the translation of research findings to athlete practices in training and competition, including practical recommendations for athletes and performance support practitioners regarding buffering agents.

## Analysis of Current Evidence

A literature search was conducted for studies published up to and including April 2023, using three online databases (PubMed, Embase and Google Scholar), with additional papers identified via the reference lists of original and review papers. For studies in females, the search terms (female OR woman OR women) AND (athlete OR sport) AND (sodium bicarbonate OR sodium citrate OR beta-alanine) were used. For extreme environmental conditions, the search terms were (heat OR altitude OR hypoxia) AND (exercise OR sport) AND (beta-alanine OR sodium bicarbonate OR sodium citrate). For combined buffering agents, the search terms were (exercise OR sport) AND (beta-alanine AND sodium bicarbonate OR sodium citrate). Inclusion criteria comprised studies investigating supplementation with buffering agents, focusing on exercise performance. Studies were excluded if they were not published in English, if there were no performance test outcomes, or if (for studies focused on females) there was no female-only or female subgroup analysis, or (for combined supplementation studies) there were multi-ingredient supplements with no isolated sodium bicarbonate, beta-alanine or sodium citrate trials. A total of 58 studies were found, comprising 29 studies in females, 18 studies in extreme environments and 11 studies in combined buffering agents. If studies were relevant to two scenarios (e.g. females and extreme environments), they were included in only one grouping, to avoid duplication of the same dataset. To standardise the allocation of studies, where duplicates occurred, the study was placed within the scenario with the lowest number of studies (Table 1).

**Table 1:** Studies included within the narrative review (*n* = 58), comprising studies in females (*n* = 29), extreme environmental conditions (*n* = 18) and combined buffering agents (*n* = 11)

Study	Year	Buffer	Highly trained?	Dose and timing	Exercise test	Familiarisation?	Dietary standardisation?	Quantified side-effects?	↑ ≥ 4 mmol/L [HCO_3_^−^]?	Performance improved in relevant performance task?
Highly trained female athletes										
Bishop and Claudius [25]	2005	SB	No	0.2 g/kg BM 110 min + 0.2 g/kg BM 50 min pre	Intermittent cycling sprint test	No	No	No	Yes	Yes
Bishop et al. [26]	2004	SB	No	0.3 g/kg BM 90 min pre	Repeated sprint cycling test	Yes	Yes	No	Yes	Yes
Delextrat et al. [27]	2018	SB	No	0.4 g/kg BM daily 3 days	Basketball simulation test	No	No	Yes	No	Yes
Durkalec–Michalski et al. [28]	2020	SB	Yes	≤ 0.1 g/kg BM daily 10 days	2 × Wingate tests	No	No	No	No	No
Edge et al. [29]	2006	SB	No	0.2 g/kg BM 90 min + 0.2 g/kg BM 30 min pre 8 weeks training	100% $\dot{V}$O_2peak_ cycling test	Yes	No	No	Yes	Yes
Gholami et al.[30]	2022	ß-A	No	6.4 g/day 4 weeks	Yo-Yo test	No	No	No	No	No
Glenn et al. [31]	2015	ß-A	No	1.6 g Acute dose	3 × Wingate tests	Yes	No	Yes	No	No
Glenn et al. [32]	2015	ß-A	Yes	3.2 g/day 28 days	Cycling time to exhaustion test	Yes	Yes	Yes	No	No
Glenn et al. [33]	2016	ß-A	No	3.2 g/day 28 days	Isokinetic strength test	No	Yes	Yes	No	No
Herda et al. [34]	2021	ß-A	No	6 g/day 3 weeks	Electromyography fatigue threshold (EM_FT_)	Yes	No	No	No	No
Karavelioglu et al. [35]	2014	SB	No	0.3 g/kg; BM 120 min pre	Yo-Yo test	No	No	No	No	No
Kozac-Collins et al. [36]	1994	SB	No	0.3 g/kg BM 120 min pre	Repeated 60 s cycling test	No	No	No	Yes	No
Kresta et al. [37]	2014	ß-A	No	6.1 ± 0.7 g/day 28 days	2 × Wingate tests	Yes	No	No	No	No
Macutkiewicz and Sunderland [38]	2018	SB	Yes	0.2 g/kg BM 180 min + 0.1 g/kg BM 90 min pre	Field hockey skill test	No	No	Yes	Yes	No
Martin et al. [39]	2023	SB	No	0.3 g/kg BM Enteric coated Individual time to peak	2000 m rowing time trial	Yes	No	Yes	Yes	Yes
McKenzie [40]	1988	SB	No	0.3 g/kg BM 120 min pre	800 m run	No	No	No	Yes	No
McNaughton et al. [41]	1997	SB	No	0.3 g/kg BM 90 min pre	60 s cycling	No	No	No	Yes	Yes
Oopik et al. [42]	2008	SC	No	0.4 g/kg BM 2 h pre + 1.5 h pre	1500 m running	No	Yes	Yes	No	No
Outlaw et al. [43]	2016	ß-A	No	3.4 g/day 4 × weekly; 8 weeks	Wingate test	No	Yes	No	No	No
Ribeiro et al. [44]	2020	ß-A	Yes	6.4 g/day 3 weeks	Running Anaerobic Sprint Test (RAST)	No	No	No	No	No
Rosas et al. [45]	2017	ß-A	No	4.8 g/day 6 weeks	Running Anaerobic Sprint Test (RAST)	No	Yes	No	No	Yes
Smith et al. [46]	2012	ß-A	No	3.2 g/day 28 days	Time to exhaustion test	No	Yes	Yes	No	No
Smith-Ryan et al. [47]	2012	ß-A	No	4.8 g/day 28 days	Time to exhaustion test	No	Yes	No	No	No
Stout et al. [48]	2007	ß-A	No	3.2 g/day; (days 1–7) 6.4 g/day; (days 8–28)	Physical working capacity at fatigue threshold (PWC_FT_)	No	Yes	No	No	No
Tan et al. [49]	2010	SB	Yes	0.3 g/kg BM 90 min pre	Water polo match simulation	Yes	No	Yes	Yes	No
Tiryaki and Atterbom [50]	1995	SC	No	0.3 g/kg BM 2.5 h pre	600 m running	Yes	No	No	No	No
Varanoske et al. [51]	2017	ß-A	No	6 g/day 28 days	Isokinetic muscle fatiguing test	No	Yes	No	Yes	No
Voskamp et al. [52]	2020	SB	No	0.3 g/kg BM 150 min pre	2000 m cycling time trial	Yes	No	Yes	No	Yes
Walter et al. [53]	2010	ß-A	No	6 g/day; (weeks 1–4) 3 g/day; (weeks 5–7)	$\dot{V}$O_2peak_ test	No	No	No	No	No
Extreme environments (heat)										
Gough et al. [54]	2021	SB	No	0.2 g/kg BM Individual time to peak	4000 m cycling time trial	No	No	Yes	No	Yes
Mündel et al. [55]	2018	SB	No	0.5 g/kg BM 3 doses at 4 h intervals	2 × Wingate tests (cycling)	No	No	No	Yes	No
Nelson et al. [56]	2008	SC	No	0.2 g/kg BM 100 min pre	62 min cycling @ ~ 60% $\dot{V}$O_2peak_	No	No	No	Yes	No
Suvi et al. [57]	2018	SC	No	600 mg/kg 960 min pre	40 km cycling time trial	No	Yes	Yes	No	No
Vaher et al. [58]	2015	SC	No	0.5 g/kg 150 min pre	5000 m running	Yes	No	Yes	Yes	No
Extreme environments (altitude)										
Deb et al. [59]	2017	SB	No	0.3 g/kg BM Individual time to peak	Critical power test	No	No	No	Yes	No
Deb et al. [60]	2018	SB	No	0.3 g/kg BM Individual time to peak	Supra-maximal 60 s cycling test	No	No	No	Yes	Yes
Fernandez-Castanys et al. [61]	2002	SC	No	0.4 g/kg BM 120 min pre	112% $\dot{V}$O_2max_ cycling test	No	No	No	No	No
Flinn et al. [62]	2014	SB	No	0.1 g/kg BM 90 min pre + 0.1 g/kg BM 60 min pre + 0.1 g/kg BM 30 min pre	4 × 120% peak power cycling	No	No	No	Yes	No
Gough et al. [63]	2018	SB	No	Trial 1: 0.2 g/kg BM Trial 2: 0.3 g/kg BM Individual time to peak	4000 m cycling time trial	No	No	Yes	Yes	Yes
Gough et al. [64]	2019	SB	No	Trial 1: 0.2 g/kg BM Trial 2: 0.3 g/kg BM Individual time to peak	4000 m cycling time trial	Yes	No	Yes	Yes	Yes
Hausswirth et al. [65]	1995	SC	No	0.4 g/kg BM 120 min pre	Isometric knee extension @ 35% maximum voluntary contraction	No	No	No	Yes	No
Kayser et al. [66]	1993	SB	No	0.3 g/kg BM 60–90 min pre	Supra-maximal cycling time to exhaustion test	No	No	No	Yes	No
Limmer et al. [67]	2020	SB	No	0.3 g/kg/day 4 days + 0.15 g/kg/day 3 days	Portable tethered sprint running (PTSR) test	No	No	No	Yes	No
McLellan et al. [68]	1988	SB	No	0.2 g/day 2 days	Incremental cycling time to exhaustion	No	No	No	Yes	No
Patel et al. [69]	2021	ß-A	No	6.4 g/day 28 days	Cycling capacity test (110% maximal power)	Yes	No	No	No	No
Robergs et al. [70]	2005	SC	No	0.2 g/kg BM 60 min pre	110% cycling $\dot{V}$O_2max_ test	No	No	No	No	No
Wang et al. [71]	2019	ß-A	No	ß-A: 6.4 g/kg/day 4 weeks	3 min maximal cycling test	No	No	No	No	No
Combined buffering agents										
Bellinger et al. [72]	2012	ß-A + SB	No	ß-A: 65 mg/day; 28 days B: 0.3 g/kg BM B: 90 min pre	4 min cycling time trial	Yes	No	No	Yes	No
Silva et al. [73]	2019	ß-A + SB	No	ß-A: 6.4 g/day; 28 days B: 0.3 g/kg BM B: 60 min pre	30 kJ cycling time trial	Yes	Yes	Yes	No	No
Danaher et al. [74]	2014	ß-A + SB	No	ß-A: 4.8 g/day; 4 weeks + ß-A: 6.4 g/day; 2 weeks B: 0.3 g/kg BM B: 90 min pre	5 × 6 s maximal cycling test	No	No	No	Yes	No
Ducker et al. [75]	2013	ß-A + SB	No	ß-A: ~ 6.0 g/day (80 mg/kg BM); 28 days B: 0.3 g/kg BM B: 60 min pre	Repeated sprint test (3 × 6 × 20 m)	No	Yes	No	Yes	No
Hobson et al. [76]	2013	ß-A + SB	No	ß-A: 6.4 g/day; 4 weeks B: 0.2 g/kg 4 h pre + B: 0.1 g/kg BM B: 2 h pre	2000 m rowing	No	No	No	Yes	Yes
Mero et al. [77]	2013	ß-A + SB	No	ß-A: 4.8 g/day; 4 weeks B: 0.3 g/kg BM B: 60 min pre	100 m freestyle swimming	No	No	Yes	Yes	No
Painelli et al. [78]	2013	ß-A + SB	No	ß-A: 3.2 g/day; 1 week + ß-A: 6.4 g/day; 3 weeks B: 0.3 g/kg BM B: 90 min pre	Trial 1: 100 m swimming Trial 2: 200 m swimming	No	Yes	No	No	Yes
Parry-Billings et al. [79]	1986	B + SC	No	B: 0.3 g/kg BM C: 0.3 g/kg BM 2.5 pre	3 × 30 s Wingate cycling tests	No	No	No	Yes	No
Sale et al. [80]	2011	ß-A + SB	No	ß-A: 6.4 g/day; 4 weeks B: 0.2 g/kg BM 4 h pre + B: 0.1 g/kg BM 2 h pre	Cycling capacity test (110% maximal power)	Yes	No	Yes	Yes	No
Saunders et al. [81]	2014	ß-A + SB	No	ß-A: 6.4 g/day; 4 weeks + ß-A: 3.2 g/day; 1 week B: 0.2 g/kg BM 4 h pre + B: 0.1 g/kg BM 2 h pre	5 × 6 s maximal cycling test	Yes	No	Yes	No	No
Tobias et al. [82]	2013	ß-A + SB	No	ß-A: 6.4 g/day; 4 weeks B: 0.5 g/kg BM/day; 7 days	4 × Wingate tests (upper body)	No	No	Yes	No	No

Studies have been arranged in alphabetical order for each scenario (females, extreme environments, combined buffering agents). In studies where more than one performance test was used, we have presented the performance test used in the analysis of studies. Performance tests were selected according to a previously published hierarchy [3]. Studies are summarised according to supplement dose and timing, and exercise test description. Studies are evaluated against their recruitment of at least highly trained (national level; tier 3) athletes as research participants, according to a recently established participant classification framework [22], the inclusion of familiarisation protocols (familiarisation trials, citation of reliability and measurement error data), pre-test dietary standardisation (citation of standardisation methods and objective data), and quantification of side-effects [5, 7, 8, 20, 21]. An increase in buffering capacity consistent with that associated with performance benefit (≥ 4 mmol/L increased blood bicarbonate concentration [HCO_3_^−^]) for extracellular buffering agents [5], and ≥ 40% increase in muscle carnosine concentration for beta-alanine studies [23]) and documented benefit in the performance tests replicating real-world competitive events (laboratory or field-based time trials, or tests replicating the profile or movement patterns required in competitive events), given the importance of these factors to athletes’ real-world performance [5, 21, 23, 24], are also reported. For combined buffering studies, the performance effect was a comparison between the combined buffering condition and the buffering agent in isolation (i.e. beta-alanine or sodium bicarbonate). Sodium bicarbonate studies are indicated by SB, SC indicates sodium citrate studies and ß-A indicates beta-alanine studies

*BM* body mass, *VO*_*2max*_ maximal oxygen consumption

Studies were analysed according to three themes:

1. Moderating factors. Studies were evaluated in terms of consistency with the current, published evidence-based guidelines for the dose and timing of supplementation, and duration and intensity of the exercise test (Fig. 1).
2. Translational factors. Female-participant-specific studies were analysed for control or standardisation of menstrual cycle (MC) status or phase and hormonal contraceptive use [83, 93], and all studies were evaluated for appropriate study design (defined as a randomised crossover with repeated measures, or matched groups design, sample size similar to previous reports, and control group) [20, 21]. Additional translational factors were the inclusion of familiarisation protocols (familiarisation trials, citation of reliability and measurement error data), pre-test dietary standardisation (citation of standardisation methods and objective data), inclusion of performance tests replicating real-world events (laboratory or field-based time trials, or tests replicating the profile or movement patterns required in competitive events) and quantification of gastrointestinal symptoms (for sodium bicarbonate and sodium citrate) and other side-effects (e.g. paraesthesia after beta-alanine supplementation) according to recently published guidelines and frameworks on females in applied sport science research, and reviews on buffering agents [5, 8, 11, 21, 83].
3. Athlete-specific factors. Studies were evaluated for the recruitment of highly trained athletes, classified as national level (tier 3) or higher according to a recently established participant framework [22], an increase in buffering capacity of ≥ 4 mmol/L [HCO_3_^−^] for extracellular buffering agents [5] and ≥ 40% increase in muscle carnosine concentration [23], and evidence of benefit in performance tests replicating real-world competitive events, given the importance of these factors to athletes’ real-world performance [5, 21, 23, 24]. For the combined supplementation studies, performance effect was a comparison between the combined buffering condition and the buffering agent in isolation (i.e. beta-alanine or sodium bicarbonate), to reflect choices that athletes and coaches may encounter when preparing for competitive events.

These factors were selected to facilitate an evaluation of the buffering studies included in this narrative review, within the specific context of their transferability to athletes’ performance, rather than a more traditional assessment of study quality. Given the narrative structure of this review and therefore the inability to objectively quantify the effect of different factors on study outcomes, we have simply reported on the presence or absence of each factor, as an indication of the rigour and/or potential for translation of the study findings.

### Highly Trained Female Athletes

Across all studies investigating buffering agents and exercise performance, only a small number have focused on female participants. Indeed, a recent audit reported that only 4% of sodium bicarbonate studies and 8% of all beta-alanine studies have included female-only research populations [11]. Despite the paucity of studies investigating the efficacy of buffering agents in females, supplement use in athletes across different sports has been reported to be similar [84] and in some cases higher than in males (e.g. 57% in females versus 47% in males [85]), which may extend to buffering agents in various athlete populations. Therefore, it is essential to quantify effects of buffering agents on female performance. There is a physiological rationale that the effects of buffering agents may differ between male and female athletes; biological differences such as the lower muscle mass and number of type II muscle fibres in females may lead to a reduced capacity for glycolysis and lower accumulation of hydrogen [H^+^] or lactate [La^−^] ions [86–90]. Furthermore, there is current interest in the potential impact of MC phase and the use of hormonal contraceptives on exercise performance in females [91, 92]. Although there is an absence of a strong link between these factors and buffering issues, changes in performance per se associated with menstrual phase/status may increase performance variability and reduce the opportunity to detect benefits due to the use of buffering agents. The potential interaction of oestrogen and progesterone with buffering agent efficacy has been poorly considered to date, with just 4% (sodium bicarbonate) and 6% (beta-alanine) of studies including female participants considering menstrual status [93]. Despite the possibility of sex-related differences in the response to the use of buffering agents, a recent summary of the few studies involving extracellular buffers in females indicated a similar increase in buffering capacity (blood [HCO_3_^−^] and pH) and performance benefit to that reported in males [19]. Nevertheless, further evaluation is required. In a recent meta-analysis, only 11 studies had an isolated female participant group for which the data could be evaluated and included in the analysis. Across the 11 studies, there were a range of different factors (e.g. participant training status, duration and intensity of exercise test) potentially impacting study findings. Within the included studies in the meta-analytic review, none quantified MC status according to the methods recently recommended within published frameworks [83, 93], and therefore it was not possible to quantify any changes associated with menstrual status or phase. Such evaluations of buffering agents in females in future research may therefore serve to integrate the effects of both extracellular and intracellular buffers, as well as to consider the moderating, translational and athlete-specific factors for buffering agents in females.

**Fig. 2:**
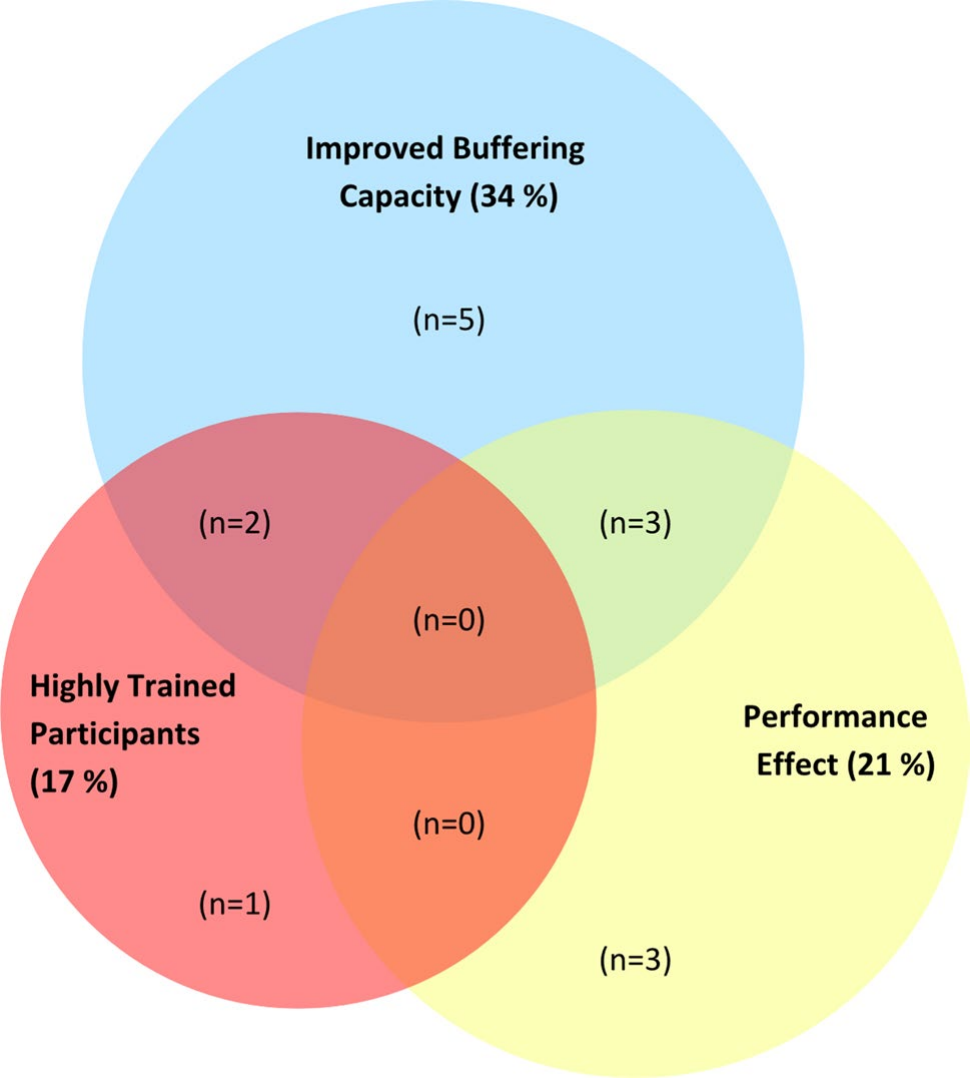
Buffering agents in females. Studies were evaluated according to their (1) recruitment of highly trained (national level; tier 3) athletes as research participants, according to a recently established participant classification framework [22], (2) an increase in buffering capacity consistent with performance benefit (≥ 4 mmol/L blood bicarbonate concentration [HCO_3_^−^] for extracellular buffering agents [5] and ≥ 40% increase in muscle carnosine concentration for beta-alanine studies [23]) and (3) a documented performance benefit in exercise tests that replicated real-world performance efforts (e.g. laboratory or field-based time trials, or tests replicating the profile or movement patterns required in competitive events). Studies that did not feature highly trained participants, evidence of performance benefit or improved buffering capacity are not included within the diagram (*n* = 15). The figure is based on the findings of 29 studies (13 sodium bicarbonate studies, 2 sodium citrate studies and 14 beta-alanine studies)

Within all the included studies on buffering agents (sodium bicarbonate, sodium citrate and beta-alanine) in females (*n* = 29), only 21% reported a performance benefit (Fig. 2). These accounted for 38% of sodium bicarbonate studies, 7% of beta-alanine studies and no sodium citrate studies. In the case of the sodium bicarbonate literature, although there was high implementation of guidelines for supplement dose (92%), over one-third (38%) used performance test intensities inconsistent with those most likely to benefit from enhanced buffering capacity [1], and therefore unlikely to be limited by acid–base disturbances. In the few studies investigating sodium citrate in females (*n* = 2), none used a supplement dose or timing that was consistent with the current evidence-based guidelines (Table 2) [1]. However, all sodium citrate studies were compliant with three design factors: implementing randomised crossover designs, adhering to pre-test dietary standardisation, and employing real-world exercise protocols (Table 3) [50, 94].

**Table 2:**
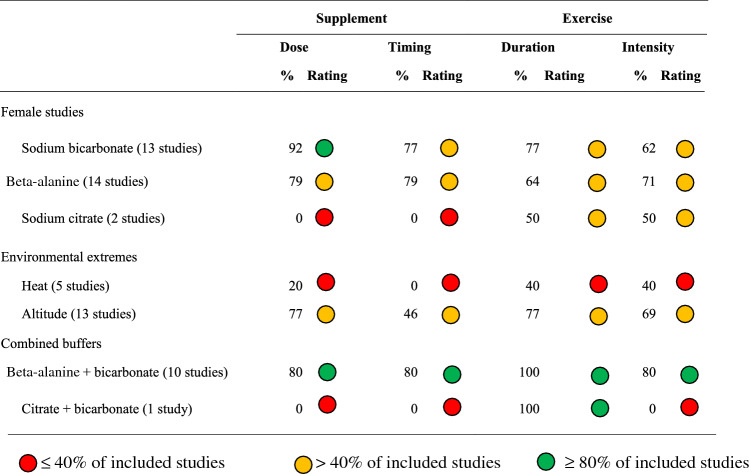
Studies in buffering agents, analysed for moderating factors (duration and timing of supplement ingestion, and duration and intensity of exercise test)

Inclusive of studies in females (*n* = 29 studies), environmental extremes (*n* = 18) and combined buffering agents (*n* = 11). Studies were evaluated according to current evidence-based recommendations for sodium bicarbonate (0.2–0.4 g/kg BM acute dose, 60–150 min prior to high-intensity exercise of 30 s–10 min duration), sodium citrate (0.5 g/kg BM acute dose, 200–240 min prior to very high-intensity exercise of 60 s–7 min duration) and beta-alanine (≥ 4 weeks chronic dose, 3.2–6.4 g/day, prior to high-intensity exercise of 30 s–10 min duration). Studies have been given a rated according to the incidence of moderating factors, with red representing ≤ 40% of included studies, yellow representing ≥ 40% of included studies and green representing ≥ 80% of included studies

*BM* body mass

**Table 3:**
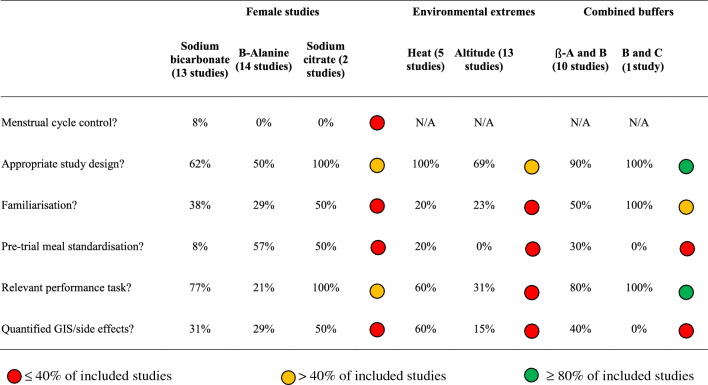
Studies in buffering agents, analysed for design factors (menstrual cycle control, study design, familisarisation, pre-trial meal standardisation, performance tasks and quantification of side effects)

Inclusive of studies involving females (*n* = 29), environmental extremes (*n* = 18), and combined buffering agents (*n* = 11). Including MC control according to recently established frameworks for female participants in sport science research [83, 93], study design (randomised crossover with repeated measures, or matched groups design, sample size similar to previous reports and control group), familiarisation trials, familiarisation protocols (familiarisation trials, citation of reliability and measurement error data), pre-test dietary standardisation (citation of standardisation methods and objective data), inclusion of performance tests replicating real-world events (laboratory or field-based time trials, or tests replicating the profile or movement patterns required in competitive events) and quantification of gastrointestinal symptoms/side-effects [5, 7, 8, 20, 21]. Coloured circles indicate the percentage of studies compliant with recommendations across total studies within (a) all buffering agents in females (sodium bicarbonate, sodium citrate and beta-alanine), (b) environmental extremes (heat and altitude) and (c) combined buffering agents. Studies have been rated based on the incidence of design factors, with red representing ≤ 40% of included studies, yellow representing ≥ 40% of included studies and green representing ≥ 80% of included studies

*ß-A* beta-alanine, *B* sodium bicarbonate, *C* sodium citrate, *GIS* gastrointestinal symptoms

Future investigations on the efficacy of buffering agents in females should focus on quantifying menstrual status, using methods to define MC phases based on hormonal profiles and/or controlling menstrual status, reporting types of hormonal contraceptive method used, tracking MC phase and standardising the phase for exercise testing and allocating participants using hormonal contraceptives to a control group [93]. Such investigations may contribute to a more thorough evaluation of the efficacy of buffering agents in female populations, particularly given that commonly reported side-effects of sodium bicarbonate and sodium citrate use (e.g. gastrointestinal side-effects) [5, 7] can be similar to symptoms experienced during menses [e.g. bloating, abdominal pain and gastrointestinal changes (stools)], and as a consequence, could be considered a confounding factor [95]. However, integration of menstrual phase control may be more difficult with studies of beta-alanine given the need for ≥ 4 weeks of chronic loading and the associated pre- and post-supplementation testing [3]. This may also apply to studies of acute sodium bicarbonate supplementation as a support for a periodised training program, with the latter only having been studied in recreationally active female athletes [29]. The recrutiment of highly trained female athletes performing exercise tests replicating real-world racing and/or competition is of high priority across all buffering supplements. Expansion of the literature in this way would enhance the translation of findings to the preparation and practice of high-performance female athletes.

### Extreme Environments

Major international events (e.g. World Championships, Olympic Games) are frequently held in hot and/or hypoxic conditions, creating challenging scenarios for athletes [14]. Performance in endurance events can be substantially impaired in hot conditions due to acute physiological responses such as decreased plasma volume [96]. While the body of evidence demonstrates a benefit in high-intensity and sprint-type exercise performance with buffering agents, there is emerging theoretical evidence that buffering agents may provide benefit in endurance exercise. This effect may be due to an accelerated glycolytic flux and increase in adenosine triphosphate production, and a reduction in the depression of muscle force due to extracellular potassium ion accumulation during demanding exercise [97–99]. Acute exposure to hot weather conditions during exercise can also lead to a greater reliance on anaerobic metabolism compared with equivalent exercise in temperate conditions, leading to an increased accumulation of [La^−^] and [H^+^] [100]. In addition to the buffering effect, pre-exercise supplementation with sodium bicarbonate or sodium citrate involves the intake of substantial amounts of sodium; this might contribute to hyperhydration (an increase in total body water above normal levels) and a reduction in the thermal and cardiovascular strain associated with exercise in the heat [101–104]. Meanwhile, training in hypoxic conditions is often used by athletes to prepare for competitions (both at altitude and at sea level), given that exposure to the low-oxygen environment may elicit physiological responses associated with the reduced oxygen availability, resulting in a decreased inspired partial pressure of oxygen (PiO_2_), and a subsequent decreased arterial oxygen partial pressure (PaO_2_) and arterial oxygen saturation (SaO_2_), contributing to a decreased maximal oxygen consumption ($\dot{V}$O_2max_) [105]. Further, an increased release of noradrenaline by the sympathetic nervous system can facilitate an increased reliance on blood glucose metabolism and increased blood lactate concentration during submaximal exercise, provided athletes are able to maintain their sea-level training pace or intensity, which has been reported with athletes who are experienced with training at altitude [15, 106]. After 2–4 weeks, adaptations occur (e.g. increased haemoglobin mass and $\dot{V}$O_2max_ at sea level), enhancing performance at sea level [106, 107]. Acute exposure to the reduced availability of oxygen at altitude results in a reduced $\dot{V}$O_2max_, and therefore the documented acute responses of hypoxia indicate that supplementation with buffering agents in the acute phase of altitude exposure may improve athletes’ capacity to maintain training intensity, as has been reported for some well trained and elite athletes during training camps at altitude [15]. Some consideration of the potential benefits of buffering agents in extreme environments has been undertaken for hot weather conditions [108] and altitude [13]. However, further interrogation of the literature on sodium bicarbonate, sodium citrate and beta-alanine supplementation in extreme environmental conditions is required to evaluate how well the available studies have accounted for modifying and translation factors.

**Fig. 3:**
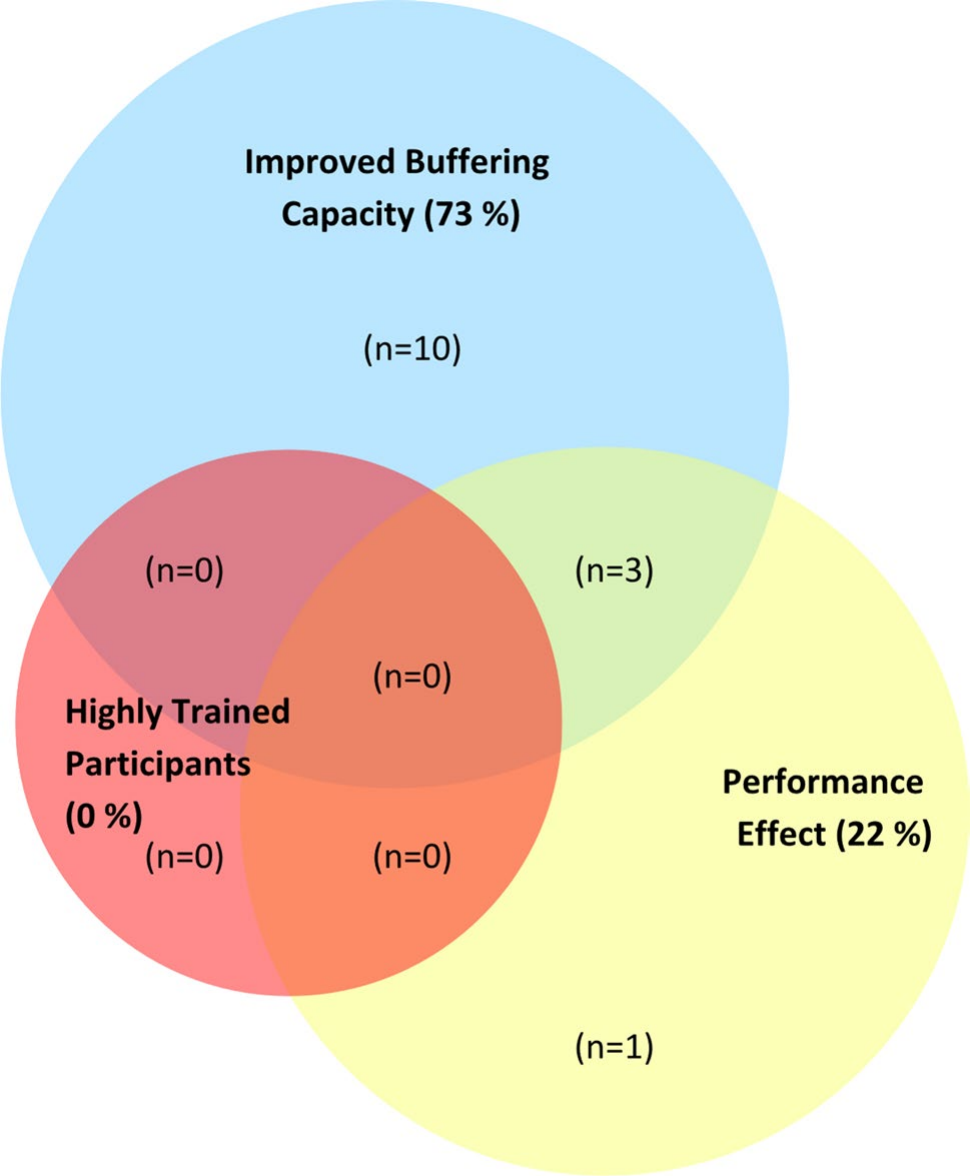
Buffering agents in altitude and heat. Studies were evaluated according to their (1) recruitment of at least highly trained (national level, tier 3) athletes as research participants, according to a recently established participant classification framework [22], (2) an increase in buffering capacity consistent with performance benefit (≥ 4 mmol/L blood bicarbonate concentration [HCO_3_^−^] for extracellular buffering agents [5] and ≥ 40% increase in muscle carnosine concentration for beta-alanine studies [23]) and (3) a documented performance benefit in exercise tests that replicated real-world performance efforts (e.g. laboratory or field-based time trials, or tests replicating the profile or movement patterns required in competitive events). Studies that did not feature highly trained participants, evidence of performance benefit or improved buffering capacity are not included within the diagram (*n* = 4). The figure is based on the findings of 18 studies (13 studies examining buffering at altitude and five studies examining buffering in the heat)

Of the 18 studies investigating buffering agents in extreme environments, five studies were in hot conditions (mean ± SD temperature 31.4 ± 2.2 °C and relative humidity 48.0 ± 8.4%) and 13 studies were in hypoxia (mean ± SD elevation 3181 ± 1024 m). Of these studies, only 22% reported a performance benefit (Fig. 3) (three altitude studies and one heat study). Within the heat literature, across the four modifying factors, ≤ 40% employed protocols consistent with the recommended use of these supplements (20% of studies for dose, none for timing, and 40% of studies for exercise duration and intensity). However, several studies conducted in hot conditions were focused on the effects of sodium bicarbonate or sodium citrate on hydration status, rather than quantifying buffering capacity and performance in the context of these environmental conditions [56–58], which may explain the lack of consistency with buffering-specific guidelines. A common limitation of the studies conducted in the heat was the absence of adequate familiarisation practice (included only in 20% of studies). No studies conducted in the heat recruited highly trained or elite athletes, and there is therefore currently very limited transferability of these research results to high performance sport. Among studies conducted at altitude, 77% used an exercise test duration consistent with current evidence-based guidelines, but with limitations associated with the translation quality of studies, this included a lack of adequate familiarisation measures (included in only 23% of studies), no studies that included pre-test dietary standardisation, poor quantification of gastrointestinal symptoms or other side-effects (15%), and a limited incidence of performance tests with relevance to real-world outcomes (31%).

A future focus on the recruitment of highly trained and/or elite athletes for studies conducted in hot and/or hypoxic conditions may increase the relevance of research findings to preparation and competition outcomes in high performance sport. Further investigation of extracellular buffering agents in hot-weather conditions is required to determine the combined effect of manipulating hydration and buffering characteristics on performance outcomes, or how each characteristic contributes to an observed performance benefit. This will build on the evidence that hyperhydration can be induced after ingestion of sodium bicarbonate and sodium citrate at the doses typically used to increase buffering capacity [104], and recently reported improvements in buffering capacity and performance in hot conditions [54]. The current literature provides very limited evidence that the use of buffering agents is beneficial to performance in athletes when they train or compete at altitude; however, greater confidence in this supplementation strategy might be provided by conducting studies which include supplement protocols with the recommended dose and timing, as well as design features that increase confidence in the study outcomes (e.g. familiarisation trials, quantifying of side-effects). An increase in real-world relevance may also be facilitated by the evaluation of buffering agents at the lower altitudes more commonly used by athletes for altitude training. Indeed, popular venues for training camps include Font-Romeu, France (1850 m); Colorado Springs, USA (1860 m) and Kunming, China (1860 m) [106]. The elevations at these venues are classified as low altitude according to the range of elevations across the altitude training literature [109]; however, physiological adaptations, including increases in haemoglobin mass, have been reported following training at altitudes of 1400 m [15], 1600 m [110] and 1800 m [111].

### Combined Buffering Agents

Studies of the supplementation practices of athletes frequently document the concurrent use of a number of different products [112, 113]. Undoubtedly, some of these practices represent indiscriminate polypharmacy, with athletes being unaware of the cumulative quantity and range of ingredients that they are ingesting [114]. However, in other situations, the athlete may use two or more supplements simultaneously with the deliberate intent of combining the separate actions of each product. Within the scientific literature, one of the most frequently investigated supplement combinations is sodium bicarbonate and beta-alanine [1, 3, 16, 24, 115]. This underscores the theoretical potential for an additive effect of extracellular buffering via increases in blood pH and [HCO_3_^−^] and intracellular buffering via increased muscle carnosine content compared with the use of either strategy alone, as well as the anecdotal reports of combined use of these two buffering agents by athletes [16]. There is some evidence that combined beta-alanine and sodium bicarbonate supplementation may elicit performance benefits [3, 115], particularly when compared with beta-alanine ingestion in isolation. Further investigation of the translation of research findings to athletes’ performance has however yet to be performed.

**Fig. 4:**
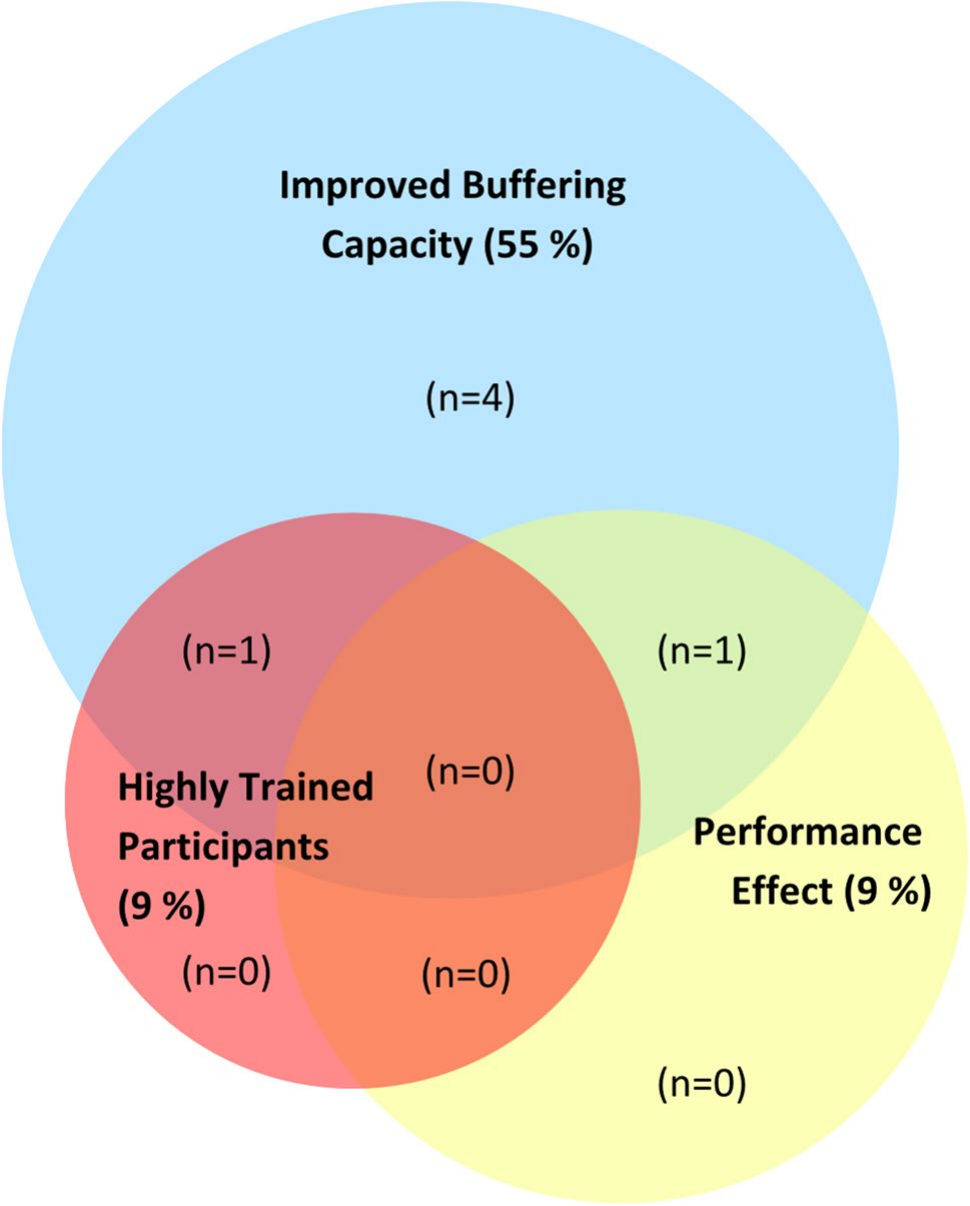
Combined buffering agents. Studies were evaluated according to their (1) recruitment of at highly-trained (national level, tier 3) athletes as research participants, according to a recently established participant classification framework [22], (2) an increase in buffering capacity consistent with performance benefit (≥ 4 mmol/L blood bicarbonate concentration [HCO_3_^−^] for extracellular buffering agents [5] and ≥ 40% increase in muscle carnosine concentration for beta-alanine studies [23]) and (3) a documented performance benefit in exercise tests that replicated real-world performance efforts (e.g. laboratory or field-based time trials, or tests replicating the profile or movement patterns required in competitive events). Studies that did not feature highly trained participants, evidence of performance benefit or improved buffering capacity are not included within the diagram (*n* = 5). The figure is based on the findings of 11 studies (10 studies investigating sodium bicarbonate and beta-alanine and one study investigating sodium bicarbonate and sodium citrate)

Of the 11 studies investigating the combination of buffering agents included in our analysis, 10 explored sodium bicarbonate and beta-alanine supplementation, and one explored sodium bicarbonate and sodium citrate supplementation. When the combined effect of beta-alanine and sodium bicarbonate supplementation was compared with beta-alanine in isolation, a performance improvement was reported in only one study (Fig. 4). This outcome was not explained by moderating factors, given that, compared with the evidence-based guidelines for both dose and timing, all studies were compliant with duration, and 73% were compliant with intensity of the exercise test guidelines. Our finding contradicts other evaluations of the literature [3, 115] which have reported a further performance benefit when sodium bicarbonate was added to beta-alanine supplementation. This discrepancy is likely due to the different analysis methods used in the current narrative review; we examined performance effects only when the exercise tests were relevant to real-world performance (8/11 studies), although studies with more controlled exercise protocols were included in the analysis of other study factors. Further, our narrative summary is different to the meta-analytical approach which can magnify the numerical outcome of studies with small samples in which the individual study failed to detect an effect. To enhance our knowledge of this area, we recommend that the combination of buffering agents be evaluated in the context of performance tests replicating real-world performance, and in highly trained participants. Further, investigation of other iterations of buffering agent combinations (e.g. beta-alanine and sodium citrate) with comparisons between the isolated buffering agents and the supplement combination is warranted. Evaluation of the efficacy of combined buffering agents in scenarios relevant to athletes’ training practices and competition preparation (e.g. altitude and/or heat training camps) is also required to determine the differentiation in buffering capacity and performance in these environments. The potential translation of such research findings to the preparation for major championship events held in hot and hypoxic conditions is relevant to the compromised buffering capacity reported when athletes are acutely exposed to these challenging environmental conditions [100, 107].

## Practical Applications and Recommendations

### Interactions of Buffering Agents with Other Supplements

Theoretically, other evidence-based supplements could be used in combination with buffering agents, in the hope of achieving an additive or synergistic effect by addressing different contributors to the fatigue or decay in exercise performance [116]. Potential benefits could occur by adding the separate effects of increased substrate availability (e.g. creatine supplementation), reduced perception of pain or effort (e.g. caffeine), or effects on muscle contractility (e.g. nitrate) [1]. Of course, a range of interactions between these supplements and buffering agents could occur, across the spectrum of negative through neutral, to synergistic [16]. Counter-productive outcomes could occur if the combination of products exacerbates the risk or magnitude of side-effects. For example, a study of well-trained rowers undertaking 2000 m rowing ergometer time trials found that the 2% improvement in performance associated with the pre-exercise intake of caffeine was negated by combining it with a bicarbonate supplementation protocol; this was attributed to the associated gastrointestinal discomfort [117]. Here, it should be noted that even if the focus was limited to the relatively few performance supplements that are individually considered to be evidence based, conventional controlled trials would be unable to adequately investigate the numerous permutations and combinations of these products with buffers on sports performance [1]. A sophisticated approach to field testing or research methodology would be needed to identify the optimal protocol for combining the use of some or all of these supplements in key sporting events.

#### Buffers and Nitrate Supplementation

Two supplements may directly interact with the extracellular alkalosis associated with the use of sodium bicarbonate/citrate. Nitrate supplementation, often in the form of consumption of beetroot juice/extracts, has been shown to enhance the performance of sports and exercise protocols [118]. Limitations of the existing literature are associated with a lack of evidence for performance benefit in well-trained athletes and females; however, the existing evidence indicates the primary mechanism is focused on downstream effects of the enhanced bioavailability of nitric oxide (NO) on exercise economy and muscle contractility [118]. The nitrate–nitrite–NO reaction provides an alternative pathway for NO production to the better known NO synthase-dependent pathway, and because of its ability to function in the presence of hypoxia and acidosis, this auxiliary source of NO availability may be particularly important during exercise because these conditions may occur locally within the muscle [119]. There is theoretical evidence that the combination of nitrate supplementation and bicarbonate supplementation protocols, each of which individually is considered to benefit the performance of sustained high-intensity (e.g. 2–8 min) sports/exercise protocols, might be counteractive if the extracellular buffering effects of the bicarbonate negate the efficacy of nitrate supplementation by interfering with one of the physiological conditions in which the nitrate–nitrite–NO pathway might provide advantages [24, 120]. Only one study has investigated this interaction, by studying the individual and combined effects of supplementation with sodium bicarbonate and beetroot juice crystals on 4 km laboratory-based cycling time trial performance [120]. Under the conditions of the study, there were no detectable effects of either or both supplements on time trial performance. Additional studies should continue to pursue the hypothesis by investigating these supplements using scenarios (e.g. choice of subjects, supplement protocols and exercise conditions) in which performance benefits are evident, and collecting physiological measures (e.g. plasma nitrate, nitrite) to quantify the response.

#### Buffers and Ketone Ester Supplementation

More recently, ketone ester supplements have been proposed to enhance endurance performance via effects associated with an exogenously derived increase in plasma ketone bodies [121, 122]. However, the majority of studies involving ketone ester supplementation pre- or during exercise have failed to show performance benefits, with some showing a decrement in exercise capacity or performance [123, 124]. As is the case with other supplements, this may reflect differences in study protocols previously mentioned and the failure of most studies to achieve the scenario in which benefits might be elicited [118, 122]. One of the potential causes of a performance impairment or interference with potential benefits of ketone ester supplementation is that the ingestion of ketone esters before or during exercise in typically used doses (e.g. 20–40 g) is associated with an increased acid load, causing a 0.05–0.10 reduction in pH and a decrease in the alkaline reserve [125]. This alteration in acid–base balance has been postulated to increase the perception of effort and contribute to the impairment of high-intensity exercise [126]. Indeed, a race-simulating cycling protocol in which 65 g of ketone ester was consumed before and during the initial phases of exercise (3 h submaximal intermittent cycling, followed by a 15 min time trial and an all-out sprint at 175% of lactate threshold), found a transient disturbance of acid base balance (reduction in blood pH and [HCO_3_^−^]) and a compensatory increase in ventilation [126]. However, the addition of a bicarbonate protocol (0.3 g/kg body mass [BM]) with the pre-exercise ketone ester supplementation protocol was able to counteract this acidosis, and was associated with a 5% increase in power output during the 15 min time trial above the control and ketone ester trial, without affecting gastrointestinal symptoms or the performance of the all-out sprint [126]. The authors of this study concluded that the use of buffering agents in association with ketone esters could provide an opportunity to ‘unlock the potential benefits of ketone ester supplements’. However, a follow-up study from the same group, in which the bicarbonate-ketone ester combination was ingested prior to a shorter exercise bout (30 min cycling time trial and all out sprint), reported that the neutralisation of the acidosis associated with the ketone ester was not able to reverse the slight decrement in performance associated with exogenous ketosis alone, and that the best performance (similar time trial performance but increase in sprint outcome) was associated with the use of bicarbonate alone. These studies highlight the complexity of metabolic scenarios achieved by the combinations of supplement protocols and real-life sporting events [127]. Although future studies may continue to fine tune such protocols and improve our understanding of the biochemistry of exercise, it is likely that they will be unable to cope with the performance aspects that arise from the stochastic nature of many real-life sports.

### Side-Effects and Safety Considerations

#### Beta-alanine

An important consideration for athletes wishing to utilise any supplement within training or competition is the presence of side-effects or risks associated with the product. There is accumulating evidence of the safety of beta-alanine supplementation when it is ingested in the protocols (dose and duration) that have been scientifically investigated. The longest study to date showed that 24 weeks of beta-alanine supplementation at 6.4 g/day did not lead to any changes in clinical markers of renal, hepatic or muscle health in healthy individuals [128]. The same study showed no reduction in the muscle taurine pool [128], which is an important consideration since animal studies have shown that beta-alanine supplementation can reduce the intracellular taurine content due to competition between beta-alanine and taurine for their shared transporter, TauT. Taurine depletion is associated with adverse health and performance outcomes in animal models [129, 130]. The original data of Saunders et al. [128] are supported by a systematic risk assessment of beta-alanine that showed no effect of supplementation on muscle taurine content in humans [131]. A further theoretical concern is that beta-alanine supplementation may reduce the intracellular content of free histidine [132], given that histidine is also required to synthesise carnosine. Experimental evidence on this is contrasting [133, 134], though meta-analytical data from humans indicate that beta-alanine supplementation does not reduce free histidine within commonly employed doses [131]. Taken together, the available evidence indicates that beta-alanine supplementation can be considered a safe ergogenic aid when taken in doses of 6.4 g/day up to 24 weeks.

Despite the clear safety of chronic beta-alanine ingestion, a common side-effect that is experienced acutely is paraesthesia, which is described as an uncomfortable prickly sensation on the surface of the skin. This sensation of paraesthesia occurs due to the binding of beta-alanine to peripheral neuronal receptors [135]. Although it can be considered an unpleasant or unwanted sensation, there is no evidence to indicate that paraesthesia is harmful per se and it should be considered a side-effect as opposed to an adverse effect. The occurrence and intensity of paraesthesia are dose related and closely relate to the time and peak of blood beta-alanine concentration [23]. Given this, strategies to slow the release of beta-alanine into the blood, thereby reducing the extent of peak blood beta-alanine, can reduce or even entirely prevent the symptoms of paraesthesia. The most common method is to split the desired dose throughout the day [23]; for example, to divide the commonly used dose of 6.4 g/day into 1.6 g every 3–4 h. This strategy may reduce, but not completely eliminate, the occurrence of paraesthesia [136]. A second option is to use sustained-release tablets; these have been shown to reduce the release of beta-alanine into the bloodstream, substantially lowering the incidence of paraesthesia in comparison to beta-alanine in rapid release format or dissolved in an aqueous solution [137, 138].

#### Sodium Bicarbonate

The unpleasant side-effects that follow sodium bicarbonate ingestion are well known and can include abdominal pain, stomach ache, flatulence, nausea, diarrhoea, vomiting and headache [139, 140]. Since these side-effects may reduce or negate the benefit of the buffering effect of sodium bicarbonate [140, 141], it is important to determine their severity, their likelihood of impairing performance and strategies to minimise/avoid their occurrence. It is well known that the occurrence and intensity of these symptoms are intrinsically linked to the dose ingested, with greater discomfort experienced as the dose increased between 0.1 and 0.5 g/kg BM [142]. Therefore, finding the lowest effective dose may be the best approach for avoiding symptoms and negative effects on exercise outcomes. While the 0.3 g/kg BM dose appears to be optimal for ergogenic effects, a lower 0.2 g/kg BM dose may be equally effective, with reduced gastrointestinal side-effects, particularly when the intake is timed to coincide with the peak circulating bicarbonate concentrations of the exercise bout [143, 144]. Unfortunately, it may not always be practical for athletes to have their individual time-to-peak determined, and there is evidence that this metric is not consistent within individuals [145]. Therefore, in the absence of a strategy to guarantee well-timed lower-dose supplementation, other methods to reduce side-effects appear warranted.

Methods to minimise/avoid the undesired side-effects associated with sodium bicarbonate ingestion have been investigated. One previous study [146] investigated a variety of protocols involving the ingestion of 0.3 g/kg BM sodium bicarbonate; variables included the use of solutions or capsules, different volumes of solution (7 or 14 mL/kg BM), different ingestion periods (30 or 60 min pre-exercise) and the presence or absence of a carbohydrate-rich meal (1.5 g/kg BM). While reported side-effects were similar between most protocols, the lowest incidence of symptoms (based on systematic quantification of symptoms using a validated scale) was reported when supplement ingestion was performed in conjunction with the carbohydrate meal. These data suggest that athletes should ingest their sodium bicarbonate dose with a small carbohydrate-rich meal to minimise side-effects. Other studies have employed different split-dose strategies to avoid side-effects, though many still report some discomfort [140, 147–149], while direct comparisons of ingesting a single bolus versus a split-dose strategy are lacking. Chronic (multi-day) consumption of sodium bicarbonate may be an effective way to increase blood bicarbonate levels without the need for supplement ingestion on the competition day itself since circulating bicarbonate may remain elevated for up to 48 h [150–152]. More recently, strategies that minimise bicarbonate interaction with the stomach acid have been suggested to minimise side-effects and optimise blood bicarbonate increases [153]. Delayed-release [154] or enteric coated capsules [139, 155] show promise in reducing the common side-effects compared with sodium bicarbonate ingestion in gelatine capsules or solution, though this research line remains in its infancy. Transdermal delivery of sodium bicarbonate has also been investigated, but there is currently insufficient evidence to suggest any benefit to performance [156]. A combination of sodium bicarbonate ingestion in enteric capsules alongside a carbohydrate meal may be an interesting, but yet unstudied, strategy.

#### Sodium Citrate

Sodium citrate is often considered an attractive alternative to sodium bicarbonate due to the perceived lower risk and intensity of side-effects [24, 157]. It is important to note, however, that side-effects do still occur with sodium citrate, the most common of which are similar to those experienced with sodium bicarbonate: stomach cramps, bloating, nausea, vomiting, urge to defecate, diarrhoea, thirst and headache [94, 158, 159]. In fact, recent evidence suggests that the side-effects experienced with the recommended dose of sodium citrate (0.5 g/kg BM) lead to the same number and intensity of symptoms as those with the recommended dose of sodium bicarbonate (0.3 g/kg BM) [160]. The prevalence and intensity of these symptoms increase in a dose-dependent manner for 0.5, 0.7 and 0.9 g/kg BM [161]. Thus, dose also appears to be a key factor when considering the incidence and intensity of side-effects with sodium citrate.

The side-effects associated with sodium bicarbonate and sodium citrate appear similar in symptoms, incidence and intensity. Nonetheless, comparison between studies is somewhat limited by the lack of a standardised method of obtaining and reporting these symptoms. Furthermore, although many reported side-effects are unlikely or less likely to substantially influence exercise performance (e.g. burping or flatulence), others may be detrimental to performance (e.g. diarrhoea or vomiting). Further, some symptoms are likely to be more problematic in some sports (e.g. running events) than others (e.g. cycling), and in some cases, athletes might well be willing to experience some of these symptoms, particularly if they are mild and if it means performance gains. The intensity of these symptoms and moment at which they are reported in relation to exercise are also key factors. Further research should strive to determine whether the combination of strategies (e.g. carbohydrate-rich meal co-ingestion and gastro-resistant capsules) that minimises symptoms leads to further reductions in their number and intensity. Athletes should experiment with these supplements throughout training and adapt the dose and timing of these supplements accordingly based upon recommendations (see Sect. 3.3) and their own experience of side-effects.

### Recommendations for Athletes and Performance Support Practitioners

The importance of supplementation strategies that are specific to individual athletes has been emphasised in the literature [1]. This is particularly relevant given the paucity of real-life considerations within published studies such as effect of sex differences, differences due to athlete calibre, use in extreme environments, and the combined use of extracellular and intracellular buffering agents. We therefore provide a summary of the current evidence in this area to develop evidence-based supplementation strategies for individual athletes, as detailed below (Table 4).

**Table 4:** Proposed model for the integration of evidence-based approaches to the development of individual buffering protocols for athletes preparing for major championship events, based on the established effect of modifying, translation and athlete-specific factors ([1, 3, 7, 16, 20, 21, 24]

Phase	Key elements
Phase 1: preparation	**Athlete selection** Established training and nutritional practices; improve nutritional literacy where feasible **Timing of supplementation trials** Adequate time available prior to major competitive events; establish specific buffering strategy (isolated/combined) Documentation of relevant details of competitive events (e.g., held in hot and/or hypoxic conditions) Buffering agent trials integrated into event preparation **Audit of supplementation practices** Documentation of current supplementation practices Identify opportunities for co-supplementation with evidence-based combination Removal of co-supplementation with limited evidence or risk of negative side-effects **Menstrual cycle information** Establish individual symptomatology patterns associated with menstrual status
Phase 2: isolated buffering trials	**Establish supplement dose and timing** Administer buffering agents according to evidence-based timing and doses Quantify gastrointestinal symptoms and other side-effects Modify supplement dose and timing within evidence-based range Further modifications as required (e.g., split doses, slow-release tablets, individual time to peak and chronic dosing for extracellular buffers)
Phase 3: buffering trials during training	**Establish training session duration and intensity** Selection of high intensity session for sodium bicarbonate and beta-alanine, and very high intensity session for sodium citrate; periodised according to chronic beta-alanine loading if relevant Quantification of buffering capacity for sodium bicarbonate and sodium citrate trials Quantify training capacity during trial training sessions
Phase 4: buffering during races/competition	**Trial under race/competition conditions** Quantify buffering capacity if feasible Quantify gastrointestinal symptoms and other side-effects Quantify race/competition performance

#### Phase 1—Preparation for Use of Buffering Agents

Within the athlete’s preparation phase, we recommend an audit of their training history and current training practices, as well as nutritional practices and supplement use. The available time that can be dedicated to experimentation and fine tuning of supplementation protocols should also be identified. It has been recommended that supplementation practices be undertaken only by athletes with an established nutritional literacy and training practices; therefore supplementation with buffering agents is unlikely to be introduced for junior or inexperienced athletes [21]. During this phase, performance support practitioners (e.g. sports dietitians) may dedicate time to improving athletes’ nutritional literacy, to enhance the athlete’s overall nutritional practices in the short and/or long term. Priority should be given to supplements, taken individually or in combination, with robust evidence of effectiveness and low risk of side-effects (e.g. taking caution with the combination of caffeine and sodium bicarbonate [117]), and the specific buffering strategy should be identified (e.g. use of sodium bicarbonate in isolation, or in combination with beta-alanine). Importantly, current and historical data on female athletes’ MC and symptoms should be collected (e.g. use of hormonal contraceptives, timing of phases [91, 92]) to establish an understanding of individual athletes’ menstrual status and associated symptomology patterns. Assessment of the suitability of introducing the use of buffering agents prior to major competitions would ideally include confirmation of adequate time and opportunity to include trials within the athletes’ periodised preparation [21]. It is anticipated that several months is likely to be required, to identify opportunities for repeated trials during suitable training sessions of high intensity (for acute sodium bicarbonate supplementation) and very high intensity (for acute sodium citrate supplementation), which will need to be coordinated with chronic loading of ≥ 4 weeks for beta-alanine trials, and/or trials of acute sodium bicarbonate or sodium citrate combined with beta-alanine.

#### Phase 2—Isolated Buffering Trials at Rest

We recommend that when athletes are deemed suitable to undertake trials of the use of buffering agents, this work is undertaken in conjunction with performance support practitioners (e.g. sport scientists and sports dietitians). Activities should focus on key modifying factors that can impact the efficacy of buffering agents (supplement dose and timing), with these first being performed under resting conditions to avoid the additional variables associated with training sessions. Evidence-based supplement dose and timing should be used (Fig. 1), supported by the collection of data that can inform the individual athlete’s responses to the use of buffering agents in methods that are practical within the specific conditions of their event [20, 21]. Validated or previously cited scales can be used to quantify gastrointestinal symptoms (most relevant for sodium bicarbonate and sodium citrate [162]), and other side-effects routinely reported during beta-alanine supplementation regimes (e.g., paraesthesia) [137]. It is valuable, where possible, for the sports scientist to monitor increases in blood [HCO_3_^−^] in conjunction with the use of extracellular buffering agents using portable blood-gas analysers. The interpretation of the combination of such data (e.g. timing of increases in buffering capacity and gastrointestinal symptoms) can be used to inform any modifications to supplement dose (e.g. decreases within the evidence-based range) and timing (e.g. increased duration prior to training sessions to avoid gastrointestinal symptoms). Additional strategies (e.g. use of split doses of beta-alanine to refine the daily dosing protocol, slow-release capsules for sodium bicarbonate and sodium citrate, and co-ingestion with high-carbohydrate foods and fluids) may provide additional benefit [1, 24].

#### Phase 3—Buffering Trials During Training

This phase provides an opportunity to implement trials of buffering agents within training sessions that provide event-specific characteristics likely to benefit from manipulation of acid–base balance (i.e. exercise of appropriate mode, duration and intensity) [1, 3, 5, 7]. It is important that these include high-intensity training sessions (for sodium bicarbonate and beta-alanine trials) and very high-intensity training sessions (for sodium citrate trials) that replicate racing or competition demands, to increase the translation of the trials to the athlete’s real-world competition experience [1, 20, 21]. These sessions should be selected according to a periodised approach to supplementation, allowing ≥ 4 weeks’ daily beta-alanine supplementation and/or adequate time on the day of the trials for the acute pre-session ingestion of sodium bicarbonate (60–150 min) or sodium citrate (200–240 min) [1, 3, 5, 7]. Another important feature of this phase is that similar sessions be conducted without supplementation, to facilitate a comparison with supplemented sessions. Consideration of factors that reduce performance variability (e.g. standardised pre-training meals that replicate the normal pre-race diet and familiarisation with training sessions performed) may add further rigour to trials conducted. Within this phase, continuation of the data collected during phase 2 (quantification of gastrointestinal symptoms, any other side-effects and extracellular buffering capacity) can inform additional modifications to supplement timing and dosing, as required. Repetition of standardised trials which quantify any changes to training capacity associated with the use of buffering agents will assist with making decisions about potential benefits during competition [1, 16].

#### Phase 4—Buffering During Races/Competitions

Additional refinement of athletes’ buffering practices can be achieved by the introduction of buffering agents (according to the individual protocols developed during phases 1–3 described above) during minor competitions in the lead-up to the major events [1]. Where feasible, it is useful for the sport scientist to quantify buffering capacity, performance metrics, and any side-effects associated with strategies. Such an approach may serve to increase the athletes’ confidence in their buffering agent protocol and reduce the likelihood of any inadvertent deleterious effects on their performance.

## Conclusion

In this narrative review, we have evaluated the current evidence for the use of buffering agents in specific populations (high-performance female athletes), extreme environments (altitude and heat) and in combined supplementation protocols (beta-alanine and sodium bicarbonate). We have also considered the moderating factors used in research studies (i.e. supplement dose and timing, and exercise test duration and intensity), factors specific to the translation of findings to real-world performance (e.g. controlling for menstrual status, performance tests replicating real-world competitive events) and other factors with a high level of specificity to athletes’ performance (e.g. recruiting highly trained or elite-level athletes as research participants). Within these contexts, the current evidence of performance improvements from the use of sodium bicarbonate, sodium citrate and beta-alanine in highly trained or elite athletes is limited; indeed, only 11/58 of the included studies (19%) reported a performance benefit. This outcome, however, may have been adversely impacted by some of the limitations within the existing literature (e.g. a very small number of sodium citrate studies in females, and few altitude studies including performance tests that replicate real-world performance efforts). We have highlighted priorities for future research, including (i) buffering studies in highly trained females, which include quantification of MC factors, (ii) buffering studies conducted at altitude with performance tests simulating real-world competitive events, and (iii) the use of various combinations of buffering agents by highly trained and elite research participants. When integrating the use of buffering agents into athletes’ preparation for major events, potential strategies include identifying opportunities for co-supplementation with other evidence-based supplements, reducing the likelihood of side-effects, and optimising key moderating factors—supplement dose and timing, and exercise duration and intensity– to develop evidence-based buffering protocols for individual athletes.

## Declarations

### Data availability

The data sets generated during and/or analysed during this narrative review are available from the corresponding author upon reasonable request.
